# Navigating the Transition to Remote Online Examinations in Undergraduate Medical Education in a Resource-Limited Setting: A Student Satisfaction and Perception Analysis

**DOI:** 10.7759/cureus.84480

**Published:** 2025-05-20

**Authors:** Sana Ali, Syed Muhammad Hammad Ali, Hamayle Saeed, Minahil Fatima Chaudhry, Javed Khalil, Noor Fatima Ahsen

**Affiliations:** 1 Department of Obstetrics and Gynecology, James Cook University Hospital, Middlesbrough, GBR; 2 Department of Medical Education, University of Central Lancashire, Preston, GBR; 3 Department of Pharmacology, Fatima Memorial Hospital College of Medicine and Dentistry, Lahore, PAK; 4 Department of Surgery, Fatima Memorial Hospital College of Medicine and Dentistry, Lahore, PAK; 5 Department of Examination, Fatima Memorial Hospital College of Medicine and Dentistry, Lahore, PAK; 6 Department of Medicine, Fatima Memorial Hospital College of Medicine and Dentistry, Lahore, PAK; 7 Department of Medicine, Joslin Diabetes Center, Boston, USA; 8 Department of Oral Biology Sciences, The Ohio State University Wexner Medical Center, Columbus, USA; 9 Department of Community Medicine, M. Islam Medical College, Lahore, PAK

**Keywords:** covid-19, online examination, online medical education, student satisfaction, undergraduate

## Abstract

Background

Remote online assessment (ROA) systems gained prominence in undergraduate medical education during the COVID-19 pandemic. This study describes a uniquely phased implementation of an ROA system in a resource-limited setting, guided by an analysis of student satisfaction and perceptions.

Methodology

This observational study presents data from the implementation of ROAs at a private medical school in Lahore, Pakistan. Four principal activities were conducted on five undergraduate medical and four dental program classes, including two mock and two summative assessments. The ROA system utilized open-source, web-based software to administer the assessments. Primary outcomes included the rate of uninterrupted exam completions and student satisfaction (measured on a five-point Likert scale) during the first three activities. Secondary outcomes examined perceptions of educational impact, perceived usefulness, and the effectiveness of anti-cheating measures.

Results

Over 800 students participated in the ROA implementation process. Clinical-year students reported significantly higher levels of satisfaction compared to preclinical students (mean ranks = 443.18 vs. 372.81, 454.31 vs. 409, and 435.50 vs. 380.13, p < 0.05), while female students consistently reported lower satisfaction. One-on-one online training sessions conducted in small groups and mini-mock exams significantly improved overall satisfaction (p < 0.005). Key challenges included insufficient exam time (n = 614, 73.9%) and internet connectivity issues (n = 470, 57%). Although live proctoring effectively deterred cheating, it also heightened exam-related anxiety (n = 313, 77.9%). Despite perceiving ROAs as inferior to traditional examinations, students acknowledged their value in supporting course completion and preparation for summative assessments.

Conclusions

A phased, adaptive approach is essential for implementing ROAs in resource-limited settings. Repeated mock exams, small-group training, and targeted support for preclinical and female students can improve satisfaction and program outcomes. Addressing technical and psychological barriers is critical to successfully integrating online assessments into undergraduate medical education.

## Introduction

Remote online assessment (ROA) systems gained prominence in undergraduate medical education (UME) as institutions adapted to the challenges posed by the COVID-19 pandemic [1]. With social distancing guidelines in place, in-person classes and clinical teaching were suspended, prompting a rapid shift to virtual learning environments using online classrooms and video conferencing tools. However, transitioning from traditional paper-based, in-person exams to ROAs presented significant challenges, particularly in low-income countries with limited digital infrastructure and resources [2].

While the pandemic catalyzed re-evaluating assessment strategies in medical education, the implementation of sustainable online assessment systems in resource-constrained settings remained difficult. Although several studies have documented adaptations to assessment methods during the pandemic, few have examined the staged implementation of ROA programs guided by student satisfaction in developing country contexts [3].

This study describes the phased implementation of an ROA system at a private medical college in a developing country, designed to replace traditional summative assessments during the pandemic. The system aimed to uphold the core principles of valid, secure, and equitable evaluation while remaining cost-effective and user-friendly [4]. Student feedback was actively incorporated at each stage to guide iterative improvements, ensuring the system remained responsive to learner needs.

This study aimed to evaluate the feasibility, effectiveness, and student satisfaction with an ROA program integrating secure digital tools, while identifying key predictors of student acceptance and educational impact.

A version of this article with preliminary findings was previously posted to the Research Square preprint server on April 26, 2022 [5].

## Materials and methods

Study design

**Figure 1:**
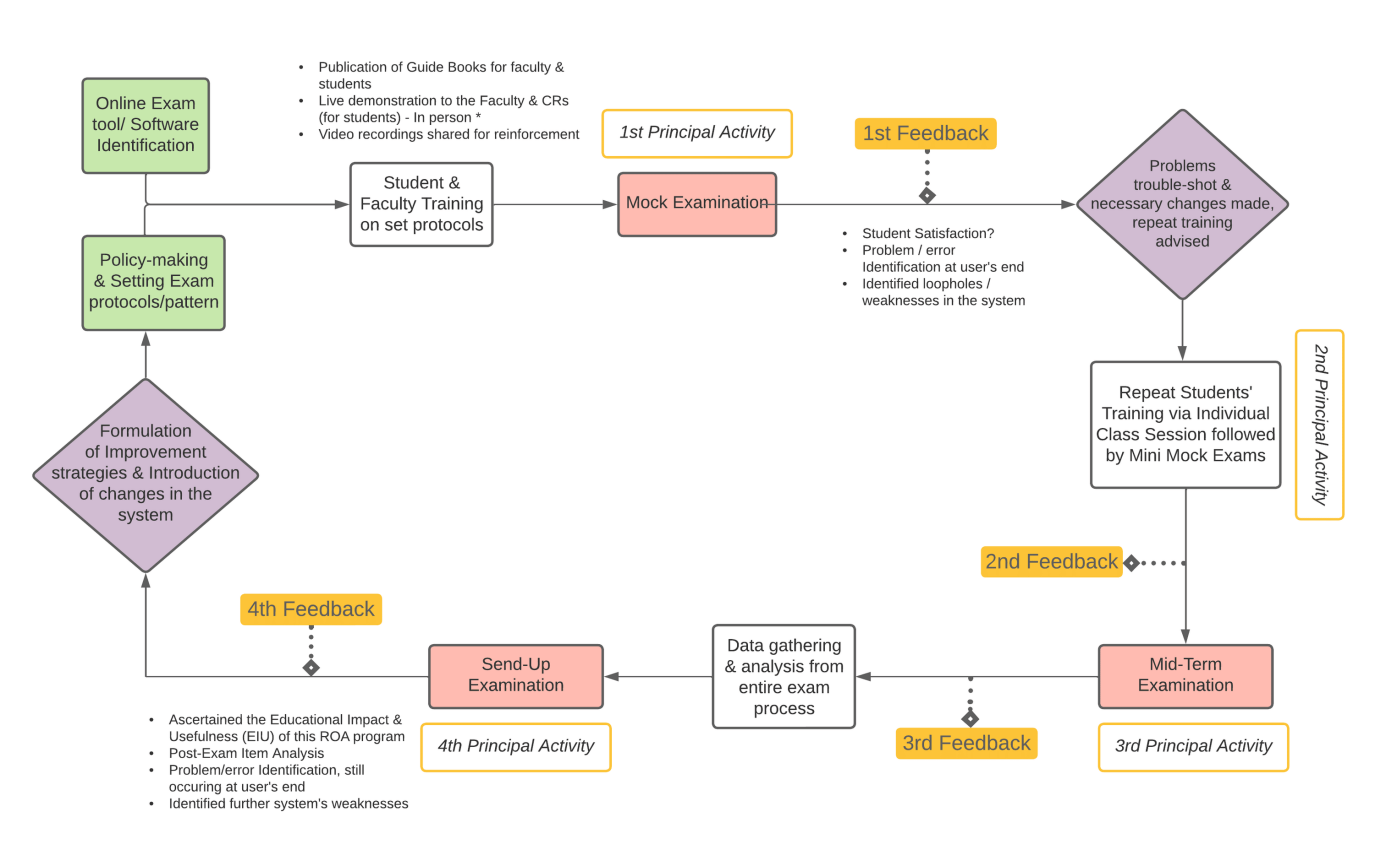
Outline of the framework for the implementation of a remote online assessment program in undergraduate medical education.

This observational study was conducted to evaluate the feasibility, effectiveness, and student satisfaction with an ROA program at Fatima Memorial Hospital College of Medicine and Dentistry, Lahore, Pakistan. The study was conducted from June to August 2020 and included the following four sequential activities: two mock examinations (for training and troubleshooting), a summative mid-term examination, and a summative send-up examination (Figure 1). Ethical approval was obtained from the Institutional Review Board of Fatima Memorial Hospital College of Medicine and Dentistry (approval number: FMH-03-2021-IRB-876-M; dated May 4, 2021), and informed consent was collected digitally.

Participants

The study included all enrolled undergraduate medical (MBBS) and dental (BDS) students who participated in all four activities through convenience sampling. Students who were absent during any of these principal activities were excluded.

Remote online assessment program and tools

The ROA program integrated the following digital tools: (1) Learning Management System: Moodle (Moodle Pty Ltd., Australia) was used for exam delivery. (2) A secure browser: Safe Exam Browser (Safe Exam Browser Consortium, Switzerland) was used with restricted access to other device functions during the exam. (3) E-proctoring: Jitsi (8×8, Inc., USA) was employed for live remote proctoring via mobile phone cameras during the final summative activity (send-up exam). For a detailed comparative research of these tools, their alternatives, utility, and compatibilities, readers are urged to refer to the work compiled by Topuz et al. [6]. The operational definitions on which our study is based are presented in a tabulated form and are validated from the literature (Table 1) [7].

**Table 1:** Operational definitions.

Terms	Definitions
Assessment or examination	An evaluation of the learning from an outlined syllabus on written, verbal, and practical formats
Summative or high-stakes	Assessments that contribute to the final grading and promotion system, e.g., term examinations, and end-of-year final examinations
Formative	Assessments carried out throughout the year to promote learning, e.g., informal class tests
Mock exam	Practice or model examination simulating a real exam situation and pattern
Mini-mock exam	Practice exams comprising fewer items than the actual exam, preceded and followed by individual class training and troubleshooting sessions
Remote	Physically away from the examination location or testing center; this may include being in a different geographic location, a different room, or a building
Online	Utilizing a computer or the internet
e-Proctoring	Virtual monitoring and invigilation of the examinees through smartphones, laptops, or portable web cameras

Study outcomes

Primary Outcomes

Primary outcomes included successful completion rates (uninterrupted exam attempts) and student satisfaction, assessed through post-exam Likert-scale surveys following the mock and mid-term assessments.

Secondary Outcomes

The secondary outcomes included educational impact and usefulness (EIU), assessed after the summative send-up exam, capturing perceptions of exam validity, motivation, and stress. Anti-cheating measures were also evaluated for effectiveness and psychological impact (among MBBS students only).

Statistical analysis

Data were analyzed using SPSS Statistics Version 22 (IBM Corp., Armonk, NY, USA) and Microsoft Excel (Microsoft Corp., Redmond, WA, USA). Descriptive statistics (percentages) summarized satisfaction levels and technical issues. Ordinal variables from Likert-scale responses were treated as non-parametric. The Kruskal-Wallis H test was used to compare rankings across groups, and multivariate regression analysis identified predictors of student satisfaction. Likert responses were collapsed into three categories (satisfied/neutral/dissatisfied) for modeling, with statistical significance set at p-values <0.05.

## Results

In total, nine classes participated in the ROA implementation process: five from the School of Medicine (first to final-year MBBS) and four from the School of Dentistry (first to final-year BDS). Overall, student satisfaction was the highest after the second mini-mock examination and individualized training session (Table 2).

**Table 2:** Comparison of clinical and preclinical students’ overall satisfaction with the remote online assessment program across different implementation stages. The data are represented as N and (%). ^a^: Following the first mock assessment; ^b^: following the second mock assessment; ^c^: following the mid-term assessment; ^d^: chi-square values generated using the Kruskal-Wallis H test; *: p < 0.05 considered statistically significant.

	Student cohort	Satisfaction level						Mean ranks	Test Statistics^d^	P-value
		Extremely dissatisfied, n (%)	Not satisfied, n (%)	Neutral, n (%)	Satisfied, n (%)	Very satisfied, n (%)	Total, n (%)			
Feedback-1^a^	Preclinical	88 (27.4)	91 (28.3)	115 (35.8)	25 (7.8)	2 (0.6)	321 (38.6)	372.81	χ² = 18.28	<0.001*
	Clinical	70 (13.7)	202 (39.6)	108 (21.2)	109 (21.4)	21 (4.1)	510 (61.3)	443.18		
	Total	158 (19.0)	293 (35.3)	223 (26.8)	134 (16.1)	23 (2.8)	831 (100)			
Feedback-2^b^	Preclinical	8 (2.5)	13 (1.4)	119 (37.8)	151 (47.9)	24 (7.6)	315 (36.0)	409.00	χ² = 7.95	0.005*
	Clinical	7 (1.3)	17 (3.0)	171 (30.5)	312 (55.7)	53 (9.5)	560 (64.0)	454.31		
	Total	15 (1.7)	30 (3.4)	290 (33.1)	463 (52.9)	77 (8.8)	875 (100)			
Feedback-3^c^	Preclinical	12 (3.8)	157 (50.0)	52 (16.6)	79 (25.2)	14 (4.5)	314 (37.9)	380.13	χ² = 12.63	<0.001*
	Clinical	10 (1.9)	220 (42.8)	54 (10.5)	177 (34.4)	53 (10.3)	21 (4.1)	435.50		
	Total	22 (2.7)	377 (45.5)	106 (12.8)	256 (30.9)	67 (8.1)	828 (100)			

In the first mock assessment, 831 of 864 (96.2%) students successfully attempted the exam, while 33 (3.8%) were unable to participate due to incompatible devices. Key challenges reported included insufficient exam time (n = 614, 73.9%) and internet connectivity issues (n = 470, 57%). Based on these insights, targeted modifications were introduced before the second mock exam (Table 3). Overall, satisfaction after the first mock was low (n = 157, 18.9%). Students from preclinical years and those who found the format non-intuitive or the allotted time insufficient were more likely to report dissatisfaction (p < 0.05).

**Table 3:** Problems identified and their solutions devised during the first mock remote online examination. The data are represented as N and (%). N = 864 (831 students were able to successfully attempt the exam). *: Based on the analysis of feedback and exam data gathered after the first mock exam (Feedback-1).

Problem(s) identified*	Solution(s) devised
Incompatible devices or software; Students unable to attempt exam (n = 33, 3.8%)	All students are encouraged to use personal computers or laptops with compatible software
Internet connectivity or speed issues (n = 470, 54.4%)	Students should arrange a standby internet source, e.g., a cell phone’s mobile data (4G), and enable students to switch network connections during the exam
Power/Electricity loss (load shedding phenomenon that is common in developing countries); students facing interruption (n = 197, 22.8%)	Split the exam into smaller components with a break time in between. Would not affect the overall exam and will allow students to make arrangements for the next exam component
Complexity of exam interface; students finding the interface less user-friendly (n = 301, 34.8%)	Make relevant changes in the exam interface to make it more user-friendly
Total time allotted for exam completion; students finding time insufficient (n = 614, 71.1%)	Give an extra time window to access the software and log in, also to account for minor delays in navigating exam questions
Access and attempt; students finding it difficult to access and attempt the exam through the laid procedure (n = 258, 29.9%)	Conduct separate live online training sessions in smaller groups and administer mini-mock exams with troubleshooting sessions afterward; address students’ problems and queries on the spot
Grievance redressal for students who will face genuine issues leading to exam disruption or rendering their exam incomplete	Formulate a mechanism to address such grievances and make an alternate mode of retaking the exam, e.g., online viva voce or a written on-campus in-person exam. Allow students to retake exams in only those components that were affected

The second mock and associated training session saw full participation (n = 875). Following this session, satisfaction significantly improved (n = 540, 61.7%). However, students from preclinical years, those who found the exam difficult to access, and those without backup internet were less likely to report satisfaction (p < 0.05).

In the summative mid-term assessment, 828 students participated and completed the feedback survey. Overall, satisfaction declined, with 323 (39%) students expressing dissatisfaction. Dissatisfaction was higher among female students, those from preclinical years, and students who did not find prior mock exams or training sessions helpful. Notably, mid-term grades did not significantly correlate with satisfaction levels.

**Figure 2:**
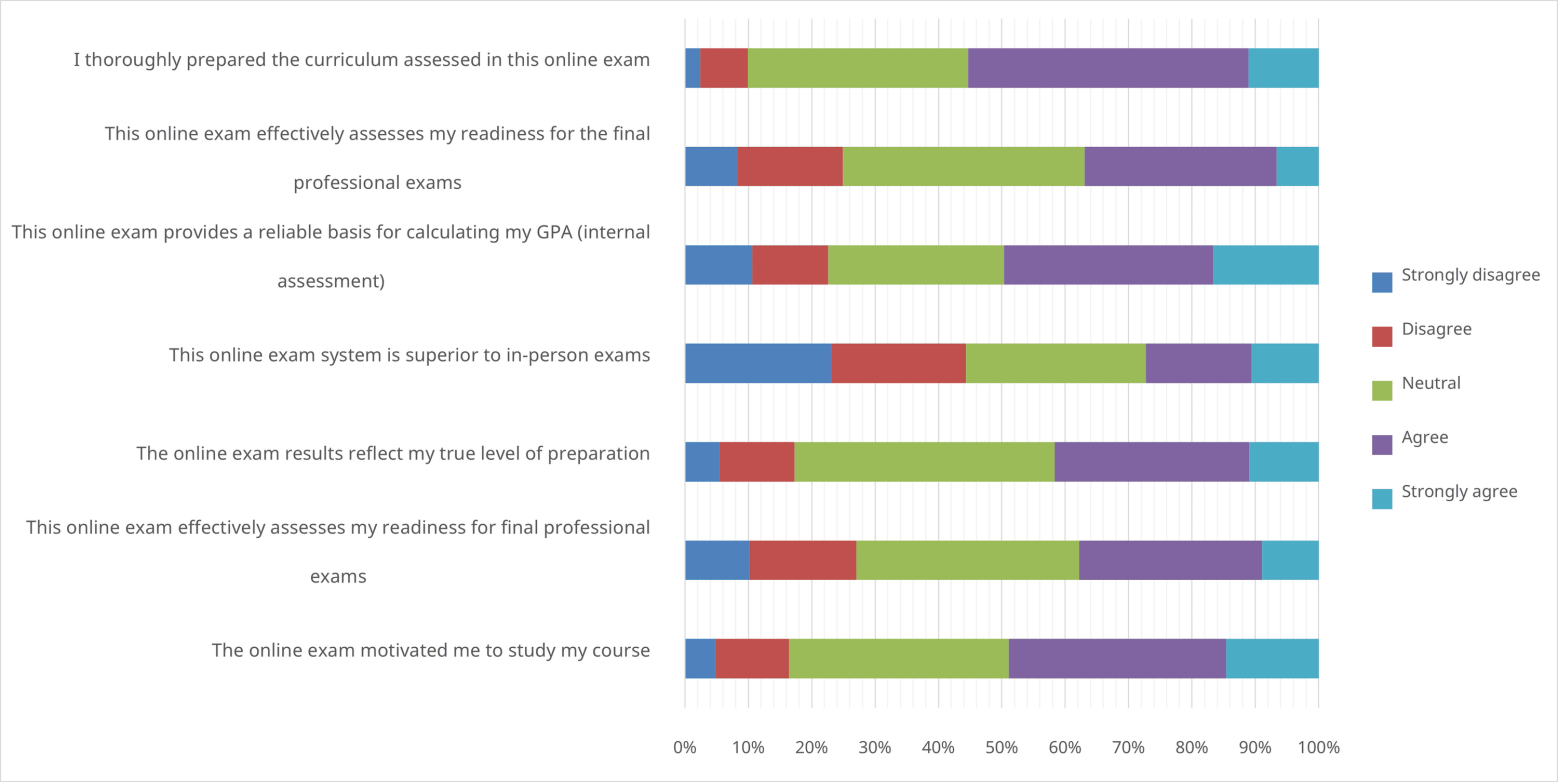
Stacked bar (100%) chart illustrating the educational impact and usefulness of the remote online assessment program.

Following the final principal activity, the summative send-up ROA, 828 students completed the EIU questionnaire. Responses were varied (Figure 2). While 367 (44.3%) students disagreed that the ROA was superior to traditional in-person exams, a majority agreed with the following statements: “I thoroughly prepared the curriculum assessed in this online exam,” “This online exam provides a reliable basis for calculating my GPA,” and “This online exam motivated me to study my course.”

Regarding anti-cheating measures, most students found the system effective in discouraging dishonest practices (n = 278, 69.2%) and encouraging exam preparation (n = 260, 64.7%). However, 28.3% (n = 114) believed exploitable weaknesses existed. A significant proportion of students reported increased stress (n = 313, 77.9%), perceived difficulty (n = 323, 80.3%), and discomfort with live proctoring (n = 308, 76.6%) (Table 4).

**Table 4:** Student’s perception on anti-cheating component of the remote online assessment program (MBBS students only). Data are represented as N and (%). The initial questions were filtered and grouped into two components through principal component analysis.

Questions	Agreement among preclinical students, n/N (%)	Agreement among clinical students, n/N (%)	Total, n/N (%)
Effectiveness of the anti-cheating system			
There are loopholes/weaknesses that can be manipulated by students	65/217 (30)	49/185 (26.5)	114/402 (28.3)
There is a need for further reinforcement to effectively curb the use of unfair means	86/217 (40)	63/185 (34.1)	149/402 (37)
The system was effective in encouraging students to prepare for summative exams	144/217 (66.4)	116/185 (62.7)	260/402 (64.7)
This system is an effective measure to discourage the use of unfair means	148/217 (68.2)	130/185 (70.3)	278/402 (69.2)
Impact on exam experience			
This system should be implemented in all upcoming online examinations	90/217 (41.5)	57/185 (30.8)	147/402 (36.6)
This system made the exam more tough to attempt	169/217 (77.9)	154/185 (83.2)	323/402 (80.3)
This system added to the overall exam-related stress and anxiety	166/217 (76.5)	147/185 (79.5)	313/402 (77.9)
Felt uncomfortable with live online invigilation (e-proctoring) through a mobile phone camera during the examination	170/217 (78.3)	138/185 (74.6)	308/402 (76.6)
Could not perform to the level of exam preparation due to the anti-cheat measures	133/217 (61.3)	113/185 (61.1)	246/402 (61.2)

## Discussion

The COVID-19 pandemic necessitated social distancing and a rapid shift from in-person to online teaching, posing significant challenges for maintaining academic assessments and examinations. One widely adopted approach was the use of open-book assessments, such as short-essay questions (SEQs) or multiple-choice questions (MCQs), administered through learning management systems [8,9]. In contrast, we implemented a more comprehensive system aimed at preserving key elements of traditional in-person examinations, particularly transparency and structural integrity, to ensure a credible summative assessment system. This allowed for uninterrupted academic progression and timely graduation of medical students, unlike open-book formats that are best utilized as formative assessments [10]. Therefore, findings from our implementation offer valuable insights into the feasibility of transitioning from traditional in-person assessments to ROAs, a shift increasingly essential for the future of medical education [11].

Our phased implementation was guided by comprehensive student feedback. Existing literature supports this approach, emphasizing that student satisfaction and feedback are critical for the successful adoption of new assessment strategies [12,13]. We found that overall satisfaction with ROAs was lower among preclinical students compared to their clinical-year peers. This key observation led to a targeted focus on supporting preclinical students, particularly those at the start of their medical education. Additional faculty members and IT specialists were designated to provide real-time troubleshooting and guidance for these students [14].

The first mock exam highlighted several technical barriers, including incompatible devices, frequent power outages (due to load-shedding), and poor internet connectivity. These challenges are common in resource-limited settings and are known impediments to the effective delivery of online education [15-17]. In our study, students without backup internet sources experienced greater disruptions, leading to incomplete or interrupted assessments.

Furthermore, the regression analysis indicated that dissatisfaction with ROAs was significantly associated with a non-intuitive exam interface and insufficient time allocation. Students particularly struggled with typing-based short-essay responses and navigating unfamiliar formats. Mohanraj et al. reported online MCQs-based assessment as a reliable predictor of students’ overall performance, citing time efficiency and accessibility as key advantages when supported by adequate technical support and anti-cheating measures [18]. However, in the context of our ROA program, we enhanced the exam interface by incorporating features like a “Wi-Fi switch” button, enabling students to change networks during exams. A single-length exam was split and modularized into three parts, which could be submitted separately, allowing for breaks in between and minimizing the impact of technical interruptions, thereby effectively incorporating SEQs as well. A redressal policy was also introduced, enabling retakes only for affected components, and additional time was granted to compensate for login or access delays.

The second training session, featuring small-group tutorials and mini-mock exams led by faculty and IT staff, proved effective. Concerns were addressed in real time, leading to a notable rise in student satisfaction. Our findings also emphasize that access to multiple internet sources, beyond just 3G/4G mobile networks, contributes significantly to satisfaction with online assessments [19].

Following the online mid-term exam, approximately 48% (n = 399) of students reported dissatisfaction, in contrast to just 5% (n = 45) after the second mock. This drop reflects anxieties regarding the effect of online summative assessments on GPA calculations. Female and preclinical students expressed more dissatisfaction, likely due to less experience with digital tools, underscoring the need for focused training [20]. Interestingly, no significant association was found between exam scores and satisfaction levels, suggesting that dissatisfaction stemmed more from systemic issues than academic performance, highlighting a potential confounder [21].

Another key challenge in transitioning to ROA was to ensure the integrity of these examinations by discouraging the use of unfair means [22]. This was the toughest challenge to address, particularly in our resource-limited setting, where technical and cultural factors were the limiting factors, with female students already showing dissatisfaction toward the ROA program. Therefore, we implemented e-proctoring in the last phase of our program when almost all other factors were accounted for and the students were well-familiarized with the basics of the ROA system.

Due to logistical constraints and the partial resumption of on-campus activities by the last phase of our ROA program, e-proctoring could only be applied to MBBS summative send-up exams. Students largely perceived the live online invigilation system as effective in deterring academic dishonesty and encouraging preparation. However, it also significantly increased stress and anxiety. These observations are consistent with existing literature, which notes that while e-proctoring can maintain academic integrity, it often exacerbates anxiety due to privacy concerns and technical hurdles [23]. Notably, stress and self-consciousness in online environments, particularly among female and domestic students, have been highlighted in recent research on diverse student perspectives [24]. For instance, camera-related anxiety during synchronous sessions (e.g., Zoom) was a recurring theme, mirroring our participants’ discomfort with the invasive nature of e-proctoring. These parallels suggest that stressors in online learning environments are multifaceted and disproportionately affect certain demographics, warranting tailored mitigation strategies.

Although most students disagreed that ROAs were superior to traditional assessments, many acknowledged that these assessments provided a reliable basis for GPA calculation and positively influenced their study habits [25]. This was crucial as summative assessments were deemed necessary besides other formats such as online quizzes, oral viva voce, case studies, and report submissions to formulate the annual GPA [26,27]. Elsalem et al. also reported overall lower rates of satisfaction among medical faculty students with ROA and their use as sole contributors to the GPA [27].

It is also worth mentioning here that while designing this ROA system, we also devised alternative plans, for instance, in case of failure of the current system, we planned to utilize open-book-type essay questions along with MCQs without e-proctoring. The option to maintain some academic integrity in such a case was through the application of sequential navigation, i.e., where every student gets a random question in a random sequence and can only navigate in one direction without an option to revisit the previous attempted question. However, this system would have heightened anxiety and further time management issues [28].

The generalizability of our findings is limited by the single-center design. Future multi-institutional studies across diverse regions could validate these results and refine remote assessment strategies. Additionally, resource constraints restricted our evaluation of student perceptions regarding anti-cheating measures, particularly the psychological impact of live proctoring. While our phased implementation (mock exams → summative assessments) identified key technical and satisfaction trends, the absence of long-term follow-up limits insights into sustained adaptability. Further research should explore longitudinal outcomes and cost-effectiveness to guide scalable implementations in resource-limited settings.

## Conclusions

Our study highlights that ROAs can sustain medical education continuity during disruptions, provided they incorporate phased training and iterative technical improvements. While preparatory interventions significantly boosted student engagement, persistent dissatisfaction among preclinical students and gender-based disparities underscore the need for tailored support. Although effective for exam integrity, live proctoring introduced notable stress, a critical trade-off for institutions to address. These findings advocate for ROA systems that balance academic rigor with equitable accessibility, particularly in resource-limited settings. Future implementations should prioritize adaptive designs that mitigate stress while maintaining assessment validity. Based on our experience and study findings, a simple exam interface with either e-proctoring (if exam integrity is crucial, e.g., in summative high-stakes assessments) or sequential navigation (in case of formative assessments designed to promote learning) will suffice. However, given the generalized dissatisfaction of students with the ROAs, educational governing and regulatory bodies should establish the validity and reliability of other non-conventional assessment tools such as open-book exams, case studies, and viva voce. Literature also suggests consistently greater student satisfaction with MCQ-based online exams and their ability to predict students’ curricular preparation and academic performance.

## Appendices

Appendix A

Table 5 presents the results of the multinomial logistic regression (MLR) analysis conducted on data from Feedback-1 (administered after the first mock assessment). Before model selection, multicollinearity among independent variables was assessed using the variance inflation factor (VIF), with all values ranging between 1 and 10, indicating acceptable levels. The proportional odds assumption for ordinal logistic regression (OLR) was tested and found to be violated (p < 0.05); therefore, an MLR model was employed as an alternative. To reduce sparsity in cell frequencies, the original five-point Likert scale for the outcome variable was collapsed into a three-point scale by merging adjacent categories. The model was then used to predict the outcome variable based on each independent variable, with other variables held constant at zero for interpretation.

**Table 5:** Multinomial regression analysis of feedback-1, after the first mock exam (sample size = 831 students). Goodness-of-fit test of overall model (likelihood ratio): Chi-square (χ^2^) = 142.88, df = 10, p < 0.005. Pseudo-R-square = 0.183. Ref: Refers to categories taken as reference in the multinomial logistic regression analysis. Odds ratio (95% confidence interval): calculated from the exponentiation of the coefficients (B). ^a^: The reference category is “Satisfied.” ^b^: Categories are merged for the ease of analysis and interpretation. The combined group “Not satisfied” is equal to “Extremely dissatisfied” plus “Not satisfied” (Likert item values 1 and 2, respectively). The combined group “Satisfied” is equal to “Satisfied” plus “Very satisfied” (Likert item values 4 and 5, respectively). *: P-value is considered statistically significant at less than 0.05.

Independent (predictor) variables	Outcome variable (students’ satisfaction)^a ^			
	Not satisfied^b^		Neutral^b ^	
	OR (95% CI)	P-value	OR (95% CI)	P-value
Gender				
Female (n = 576)	1.17 (0.78-1.75)	0.456	2.01 (1.24-3.29)	0.005*
Male (n = 255)	Ref		Ref	
Professional year				
Preclinical (n = 321)	3.00 (1.88-4.81)	0.000*	4.84 (2.90-8.08)	0.000*
Clinical (n = 510)	Ref		Ref	
Did you find the exam format a user-friendly?				
No (n = 530)	3.21 (2.17-4.75)	0.000*	4.24 (2.67-6.73)	0.000*
Yes (n = 301)	Ref		Ref	
Do you find the allotted time for exam sufficient?				
No (n = 614)	1.91 (1.25-2.90)	0.003*	1.32 (0.81-2.13)	0.264
Yes (n = 217)	Ref			
Did you face an internet speed or connectivity issue?				
No (n = 361)	1.90 (1.26-2.87)	0.002*	3.03 (1.90-4.82)	0.000*
Yes (n = 470)	Ref		Ref	

Appendix B

Table 6 presents the regression analysis based on Feedback-2 (collected after the second mini-mock assessment). Multicollinearity among the independent variables and their respective dummy variables was evaluated using VIF, which ranged between 1 and 10, indicating acceptable levels. The proportional odds assumption for OLR was tested and not violated (p > 0.05), supporting the appropriateness of the OLR model for analysis. To obtain interpretable odds ratios [Exp(B)], the analysis was conducted using the generalized linear model (GLM) framework in SPSS. To minimize sparse data and reduce empty cell frequencies, the original five-point Likert outcome variable was collapsed into three categories by combining adjacent responses. The regression model was used to predict the outcome variable for each independent variable while holding all other variables constant at zero.

**Table 6:** Ordinal logistic regression analysis of feedback-2, after the second mock exam (sample size = 875 students). Goodness-of-fit test of overall model (likelihood ratio): chi-square (χ^2^) = 150.60, df = 5, p < 0.005. Pseudo-R-square = 0.128. Ref: Refers to categories taken as reference in the ordinal logistic regression analysis. Odds ratio (95% confidence interval): Calculated from the exponentiation of the coefficients (B). ^a^: The reference category is “Satisfied.” ^b^: Categories are merged for the ease of analysis and interpretation. The combined group “Not satisfied” is equal to “Extremely dissatisfied” plus “Not satisfied” (Likert item values 1 and 2, respectively). The combined group “Satisfied” is equal to “Satisfied” plus “Very satisfied” (Likert item values 4 and 5, respectively). *: P-value is considered statistically significant at less than 0.05.

Independent (predictor) variables	Outcome variable (students’ satisfaction)^a ^	
	OR (95% CI)	P-value
Gender		
Female (n = 590)	0.90 (0.67-1.22)	0.491
Male (n = 285)	Ref	
Professional year		
Preclinical (n = 315)	0.69 (0.52-0.92)	0.011*
Clinical (n = 560)	Ref	
Did you find it easy to access the exam?		
No (n = 38)	0.07 (0.04-0.14)	0.000*
Yes (n = 837)	Ref	
Did you have a standby alternate source of internet?		
No (n = 44)	0.20 (0.11-0.38)	0.000*
Yes (n = 831)	Ref	
Was your alternate source of internet the mobile phone data?		
No (n = 536)	1.16 (0.88-1.55)	0.297
Yes (n = 339)	Ref	

Appendix C

Table 7 presents the regression analysis based on Feedback-3 (collected after the mid-term summative assessment). Multicollinearity among independent variables and their corresponding dummy variables was assessed using VIF, which fell within an acceptable range (1-10). The proportional odds assumption was tested and found to be violated (p < 0.05), necessitating the use of an MLR model instead of OLR. To address data sparsity and reduce the occurrence of empty cells, the original five-point Likert scale was collapsed into three categories by merging relevant adjacent responses. The MLR model was applied to predict the dependent variable based on each independent variable, while holding all other variables constant at zero.

**Table 7:** Multinomial regression analysis of feedback-3, after the mid-term exam (sample size = 828 students). Goodness-of-fit test of overall model (likelihood ratio): Chi-square (χ^2^) = 67.015, df = 8, p < 0.005. Pseudo-R-square =  0.090. Ref: Refers to categories taken as reference in the multinomial logistic regression analysis. Odds ratio (95% confidence interval): Calculated from the exponentiation of the coefficients (B). ^a^: The reference category is “Satisfied.” ^b^: Categories are merged for the ease of analysis and interpretation. The combined group “Not satisfied” is equal to “Extremely dissatisfied” plus “Not satisfied” (Likert item values 1 and 2, respectively). The combined group “Satisfied” is equal to “Satisfied” plus “Very satisfied” (Likert item values 4 and 5, respectively). *: P-value is considered statistically significant at less than 0.05.

Independent (predictor) variables	Outcome variable (students’ satisfaction)^a ^			
	Not satisfied^b^		Neutral^b ^	
	OR (95% CI)	P-value	OR (95% CI)	P-value
Gender				
Female (n = 556)	1.50 (1.09-2.05)	0.012*	2.20 (1.31-3.70)	0.003*
Male (n = 272)	Ref		Ref	
Professional year				
Preclinical (n = 314)	1.68 (1.21-2.32)	0.002*	1.85 (1.15-2.99)	0.011*
Clinical (n = 514)	Ref		Ref	
Do you find the mock exams and training sessions helpful in attempting this formal exam?				
No (n = 62)	5.67 (2.18-14.76)	0.000*	13.81 (4.97-38.39)	0.000*
Yes (n = 766)	Ref		Ref	
Grades scored in the mid-term exams				
	1.49 (0.47-4.70)	0.499	0.64 (0.12-3.39)	0.597
